# Expression of Aquaporin 4 and Breakdown of the Blood-Brain Barrier after Hypoglycemia-Induced Brain Edema in Rats

**DOI:** 10.1371/journal.pone.0107022

**Published:** 2014-09-29

**Authors:** Jiangshan Deng, Fei Zhao, Xiaoyan Yu, Yuwu Zhao, Dawei Li, Hong Shi, Yongning Sun

**Affiliations:** 1 Department of Neurology, Shanghai Jiao Tong University Affiliated Sixth People's Hospital, Shanghai, China; 2 School of Pharmacy, Shanghai Jiao Tong University, Shanghai, China; 3 Department of Traditional Chinese Medicine, Shanghai Jiao Tong University Affiliated Sixth People's Hospital, Shanghai, China; Medical University Vienna, Center for Brain Research, Austria

## Abstract

**Background:**

Hypoglycemia-induced brain edema is a severe clinical event that often results in death. The mechanisms by which hypoglycemia induces brain edema are unclear.

**Methods:**

In a hypoglycemic injury model established in adult rats, brain edema was verified by measuring brain water content and visualizing water accumulation using hematoxylin and eosin staining. Temporal expression of aquaporin 4 (AQP4) and the integrity of the blood-brain barrier (BBB) were evaluated. We assessed the distribution and expression of AQP4 following glucose deprivation in astrocyte cultures.

**Results:**

Brain edema was induced immediately after severe hypoglycemia but continued to progress even after recovery from hypoglycemia. Upregulation of AQP4 expression and moderate breakdown of the BBB were observed 24 h after recovery. In vitro, significant redistribution of AQP4 to the plasma membrane was induced following 6 h glucose deprivation.

**Conclusion:**

Hypoglycemia-induced brain edema is caused by cytotoxic and vasogenic factors. Changes in AQP4 location and expression may play a protective role in edema resolution.

## Introduction

With the increasing incidence of diabetes, and subsequent practice of intensive glycemic therapy, hypoglycemia has become an emerging clinical concern. Because hypoglycemia-associated autonomic failure in diabetes induces defects in glucose counter-regulation and hypoglycemia awareness, there is a high risk that severe hypoglycemia may remain undetected [1]. However, despite being a more severe clinical insult, hypoglycemia has received significantly less attention from patients and medical workers than hyperglycemia. Glucose is the fuel necessary for normal brain activity. Once the supply of glucose is interrupted, mild brain dysfunction or irreversible brain injury can occur immediately [2], [3]. Severe hypoglycemia may result in seizures, coma, and even death. Brain edema is a common pathophysiological consequence of severe hypoglycemia. Previous studies on brain ultrastructures have demonstrated that dendrite edema can occur within as little as 10 min of onset of hypoglycemia-induced isoelectric encephalography (iso-EEG) [4]. Although other reports have verified that hypoglycemia can induce brain edema [5]–[7], the mechanisms of edema formation and resolution remain unknown.

Aquaporin 4 (AQP4), the main aqueduct in the brain, has emerged as an important target for *in vivo* and *in vitro* research focused on brain edema caused by ischemia [8], [9], intracerebral hemorrhage [10], brain trauma [11], hyponatremia [12], and hepatic encephalopathy [13]. AQP4 is expressed in astrocytes and forms the main channel that quickly transports water across the membrane, in both directions. Thus, AQP4 is responsible for the development of cell edema, by introducing water into the cell, as well as edema resolution, by transporting water out of the cell. We wanted to know whether AQP4 plays a role in hypoglycemia-induced brain edema. To date, the expression of AQP4 during hypoglycemia has not been studied. In addition, the blood-brain barrier (BBB) regulates substance exchange between the periphery and central nervous system (CNS). Brain edema does not result in brain swelling until molecules such as Na^+^, Cl^−^, and water are transported across the capillaries of the BBB into the parenchyma [14]. Interendothelial tight junctions (TJ) control the paracellular diffusion of water-soluble substances from blood vessels to the brain. The disruption of these tight junctions is the direct cause of brain edema. Whether the breakdown of the BBB contributes to hypoglycemia-induced brain edema needs to be determined.

In this study, we verified a model of hypoglycemia-induced brain edema. We then studied the expression of AQP4 at the transcriptional and protein level at different time points after hypoglycemia. We demonstrated disruption of the BBB and tight junction proteins, and we conducted an *in vitro* study that showed selective AQP4 staining in the astrocytic plasma membrane after glucose deprivation.

## Materials and Methods

### Ethics statement

The animal study proposal was approved by the Institutional Animal Care and Use Committee of Shanghai Jiao Tong University Affiliated Sixth People's Hospital (Permit Number: SYXK [Hu] 2011-0128). All experimental procedures involving rats were performed in accordance with the regulations for the Administration of Affairs Concerning Experimental Animals approved by the State Council of People's Republic of China. Animals were deeply anesthetized with isoflurane prior to decapitation to minimize suffering.

### Induction of severe hypoglycemia in rats

As previously described, severe hypoglycemia was induced by a single insulin injection, with some modifications [15]. An intraperitoneal injection of 10 IU/kg regular insulin (Novolin-R, Novo Nordisk, Denmark) was administered to overnight fasted male Sprague-Dawley rats (200–280 g). Forty minutes later, rats were anesthetized with 3% isoflurane in oxygen. Anesthesia was maintained using a mask with 1.5% isoflurane. Two needle electrodes were inserted beneath the parietal scalp and the reference needle was placed in the neck muscle. A femoral vein catheter was connected with a syringe infusion pump. After the operation, atropine (1 mg/kg) was injected into the femoral muscle to inhibit respiratory secretion. Rats were ventilated with a small animal respirator (70 breaths/min, tide volume: 6 mL). Electroencephalograms were monitored using ADInstruments PowerLab data acquisition system (Labchart 8.0). For muscle relaxation, suxamethonium chloride was infused through femoral vein tubing (2 mg/h, in a volume of 0.4 mL/h). Blood sugar was detected from the tail vein using an analyzer (Roche Performa). Core temperature was maintained at 36–37.5°C using a heat lamp. Once iso-EEG was detected, isoflurane was adjusted to 2% to correct for rising blood pressure. Animals in the hypoglycemic group were sacrificed 60 min after the onset of iso-EEG. Other animals were administered an intravenous injection of 200 µL of 50% glucose to terminate hypoglycemia, and infused with 50% glucose (1.5 mL/h) until the insulin effect waned. These recovered animals were sacrificed, independently, 6 h, 24 h and 3 d after the termination of hypoglycemia. Normal control animals were fasted overnight without any other treatment. Sham hypoglycemia refers to animals that were infused with glucose immediately following the initial insulin injection to maintain normal blood sugar levels (5–10 mM) for 5 h.

### Tissue collection and immunohistochemistry

Animals from normal control and recovery groups were anesthetized with isoflurane and sacrificed at indicated time points. Each brain was divided into two hemispheres, through the midline. One-half of the parietal cortex was for water content measurement. The other half was for reverse transcription polymerase chain reaction (RT-PCR) quantification and western blot analysis. For histological analysis and immunofluorescence staining, animals were perfused transcardially with 100 mL of 0.01 M phosphate-buffered saline (PBS) followed by 250 mL of precooled 4% paraformaldehyde in phosphate-buffered solution (PB, 0.1 M, pH 7.4) for 1 h. Brains were placed in the same fixative solution for 24 h at 4°C, and then cryoprotected in a series of sucrose solutions (10%, 20%, 30% in 0.1 M PB; 4°C). Coronal sections (20 µm) were cut on a freezing cryostat (Leica, CM1900). For hematoxylin and eosin (HE) staining, 8-µm sections were cut and stained. For transmission electron microscopy (TEM), cortical tissues were postfixed in glutaraldehyde and osmium tetroxide (OsO_4_), and embedded in Epon. Tissue was then stained with uranyl acetate and lead citrate, and imaged using TEM (Philips CM120).

### Measurement of brain cortical water content

Water content of the parietal cortex was evaluated to verify the presence of hypoglycemia-induced brain edema. After decapitation, brains were removed quickly and 100 mg tissue of parietal cortex was cut and weighed in pre-weighed aluminum foil (wet weight). Tissue was then dried in an oven at 80°C for 72 h and reweighed (dry weight). Brain water content (%) was calculated as (wet weight − dry weight)*100/wet weight.

### Evaluation of BBB permeability

Evans blue (2%) was injected intravenously 30 min before the termination of iso-EEG, when rats were in a coma. Rats from the recovery groups were re-anesthetized and the Evans blue allowed to circulate for 30 min. Rats were then perfused transcardially with 100 mL of PBS to remove the blood and Evans blue from the vessels. Coronal sections were cut and photographed using a Cyber-shot camera (Sony, DSC-W390).

### Immunofluorescence staining

After rinsing twice with 0.01 M PBS for 10 min, slices were permeabilized in 0.2% Triton X-100 for 1 h, blocked with 10% goat serum with 0.3% bovine serum albumin for 1 h at room temperature, and then incubated with rabbit poly anti-AQP4 antibody (1∶200, H-80, Santa Cruz Biotechnology, USA) and mouse monoclonal anti-glial fibrillary acidic protein (GFAP) antibody (1∶200, G3893, Sigma, USA) at 4°C overnight. Slices were washed with PBS, and then incubated in a secondary antibody mixture of FITC 488-conjugated goat anti-rabbit and DyLight 594-conjugated goat anti-mouse (Jackson, USA). Subsequently, slices were counterstained with 4′,6-diamidino-2-phenylindole (DAPI) and mounted with SlowFade Light Antifade (Molecular Probes, Invitrogen, Carlsbad, CA, USA). Controls were made by omitting primary antibodies from the incubation process. All slices were scanned using a laser confocal microscope, with all images taken using the same parameters at three levels in the Z axis, with 1-µm layer spacing between images. Image Z-stacks from each section were processed into a single image using SP8 confocal software.

Cell cultures were fixed and permeabilized with 0.2% Triton X-100 for 15 min, then blocked with 5% goat serum/PBS (0.01 M) for 1 h. Cultures were incubated with rabbit poly anti-AQP4 (1∶400) and mouse monoclonal anti-GFAP (1∶200) at room temperature for 2 h, then incubated with FITC 488-conjugated goat anti-rabbit and DyLight 594-conjugated goat anti-mouse (1∶1000) at room temperature for 1 h. Slides were analyzed using the same laser confocal microscope.

### RT-PCR quantification

Total RNA was purified from 100 mg of rat brain cortical tissue using Trizol (Invitrogen, Carlsbad, CA, USA) and treated with DNase to remove any possible genomic DNA contamination. First-strand cDNAs were synthesized from 1 µg total RNA using an RT-PCR kit (Thermo Fisher Scientific, USA) according to the manufacturer's instructions. A 4 µL of aliquot of total RNA was reverse transcribed into first-strand cDNA in 25 µL of reaction mix containing 1 µL of Oligo(dt)_18_ at 37°C for 1 h and terminated at 85°C for 5 min. The RT products (2 µL) were used as templates for PCR amplification in 50-µL reactions that included the following gene-specific primers: rat AQP4 aqp4-S 5′- CTGCTAATGCTTCCCATGAC-3′, aqp4-AS 5′-GCTACCTTGCACCTTATCTG-3′; and rat glyceraldehyde 3-phosphate dehydrogenase (gapdh)-S 5′- GTCGGTGTGAACGGATTTG-3′, gapdh-AS 5′- TCCCATTCTCAGCCTTGAC-3′. Real-time PCR reactions were set up with the following: 32.5 µL of SYBR Green mix, 0.5 µL of each primer, 2 µL of cDNA template, and 14.5 µL of ddH_2_O. The mixture was incubated at 95°C for 10 min prior to PCR. The thermal cycling conditions were as follows: 95°C for 15 s and 60°C for 45 s for 40 cycles, terminated at 95°C for 15 s, 60°C for 1 min, 95°C for 15 s, and 60°C for 15 s. Reactions were run in triplicate and analyzed using ABI Prism 7300 SDS Software. GAPDH was used as a housekeeping gene. The fold increase relative to control samples was determined using the 2^−ΔΔC^_T_ method.

### Western blot analysis

Tissue or cells were homogenized in ice cold radioimmunoassay precipitation buffer (50 mM Tris-HCl, 150 mM NaCl, 0.1% sodium dodecyl sulfate (SDS), 1% NP-40, and 0.5% sodium deoxycholate) with 1% protein cocktail and 1% phenylmethylsulfonyl fluoride (PMSF) using an electrical homogenizer at 8000 rpm for 30 s. After lysis for 30 min on ice, the samples were centrifuged at 10 000 *g* for 5 min. The protein content of the supernatant was measured using a BCA Protein Assay Kit (Beyotime Biotechnology, China). To detect AQP4, each lane was loaded with 20 µg of protein and subjected to SDS-polyacrylamide gel electrophoresis on a 10% gel. To detect zonula occludens-1 (ZO-1) and occludin, 150 µg of protein was loaded in each lane and separated using electrophoresis on a 7.5% gel. Proteins were then transferred to a nitrocellulose membrane (Pall, USA), blocked with 5% skimmed milk for 1 h at room temperature, and incubated with polyclonal rabbit antibodies against AQP4 (1∶400, Santa Cruz Biotechnology, USA), occludin (1 µg/mL, Invitrogen, Carlsbad, CA, USA) and ZO-1 (1 µg/mL, Invitrogen, Carlsbad, CA, USA). After washing with TENT buffer (50 mM Tris-Cl [pH 8.0], 2 mM EDTA, 150 mM NaCl, 0.05% Tween 20), the membrane was incubated with a mixture of two secondary antibodies (an anti-rabbit IRDye-700 nm and an anti-mouse IRDye-800 nm, Roche, Basel, Switzerland). β-Actin served as the normalization control. After washing, the membrane was analyzed using an infrared scanner (Odyssey, LI-COR Bioscience, Lincoln, NE, USA). The intensity of the immunofluorescence was measured using Quantity One software.

### Cell culture

Primary cultures of astrocytes were isolated from the cortices of day old neonatal rat pups as previously described [16]. Astrocytes were cultured in a T25 culture flask with Dulbecco's Modified Eagle Medium 25 (glucose 25 mM DMEM25, Invitrogen), 100 U/mL penicillin, 100 mg/mL streptomycin, 10% fetal calf serum (FCS) and maintained at 37°C in 5% CO_2_, with a change of media every other day. After being shaken at 200 rpm for 18 h at 37°C to remove microglia and oligodendrocytes, astrocytes were plated in 10-cm diameter dishes containing DMEM5.5 (glucose 5.5 mM) with 10% FCS. Four-week-old astrocytes were used in this study.

### *In vitro* glucose deprivation (GD)/glucose reinfusion (GR)

Culture media was discarded. Cells were rinsed twice with DMEM0 (no glucose) and incubated in fresh DMEM0 and DMEM1 (glucose 1 mM) at 37°C in 5% CO_2_. For control cultures, media was replaced with DMEM5.5 immediately after washing with glucose-free DMEM. After 6 h of glucose deprivation, cells were reincubated in DMEM5.5 and harvested after 4, 8, 16, or 24 h.

### Measurement of cell injury

Cell injury was evaluated by measuring lactate dehydrogenase (LDH) activity after glucose deprivation and 4, 8, 16, and 24 h of glucose reinfusion. The LDH kit (Beyotime Biotechnology, China) was used according to the manufacturer's instructions. The ratio of LDH activity in the media to total cellular LDH was calculated as the indicative cell injury. Morphological changes in astrocyte cultures were examined using phase contrast microscopy.

### Statistical analysis

Results are presented as the mean ± standard deviation. One-way analyses of variance (ANOVA) were performed, and post hoc Dunnett's and Holm–Sidak's multiple comparison tests were used to confirm significant differences. P<0.05 was considered statistically significant.

## Results and Discussion

### Altered EEG during insulin-induced profound hypoglycemia

**Figure 1:**
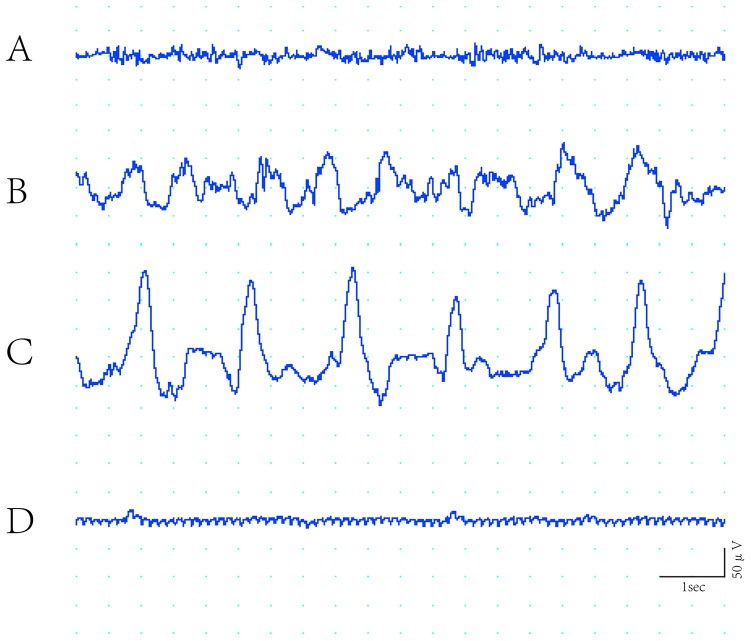
Typical changes in EEG during insulin-induced severe hypoglycemia. A. Normal EEG. B. EEG recorded 1.5 h after insulin injection showing a decrease to θ and δ with increasing amplitude. C. EEG recorded 2 h after insulin injection, bursts of δ with total suppression, the harbinger of the iso-EEG. D. Isoelectric EEG with some artifact.

The prerequisite for hypoglycemic brain damage is the onset of iso-EEG, not the level of blood sugar [2]. A typical temporal change in EEG was evoked after insulin injection (Fig. 1). Approximately 2 h after insulin treatment, iso-EEG was induced when tail vein blood sugar was approximately 0.6 mM. Here we maintained the iso-EEG period over 60 min in order to induce severe hypoglycemia.

### Increased cortical water content during hypoglycemia

We demonstrated a significant increase in parietal cortex water content during 60 min of profound hypoglycemia. A significant increase in water content was found in rats from the hypoglycemia group compared to rats that experienced sham hypoglycemia (p<0.01) and normal controls (p<0.01, Table 1). To exclude the potential lingering effects of insulin during the recovery period, we set up a sham hypoglycemia + recovery 24 h group as a control. The rats of the hypoglycemia + recovery 24 h group showed significantly increased brain water content compared to the sham hypoglycemia +24 h recovery group (p<0.01, Table 1). The water content of the sham hypoglycemia group and control group were not significantly different (p>0.05, Table 1). Previous research has suggested that hypoglycemic brain injury is not solely due to fuel deprivation, but could also be a result of glucose reinfusion [2], [17]. In the present study, we found that brain edema was not resolved after 24 h of recovery but that it had progressed. Gisselsson reported that brain edema occurred during hypoglycemia, but resolved within 3 h of recovery [7]. This is inconsistent with our results. We propose that we induced a more severe form of hypoglycemia, with an iso-EEG of 60 min, in our model; this severe form was aggravated by glucose reinfusion, which resulted in progressive edema.

**Table 1:** Measurment of brain cortical water content of hypoglycemic rats.

Group	Number of rats	Water content (%)
**Normal Control**	5	80.24±0.40
**Sham Hypo**	5	79.93±0.41
**Hypo**	7	81.28±0.75^#^
**Sham Hypo + R 24 h**	5	79.82±0.35
**Hypo + R 24 h**	5	81.62±1.08^##^
**Hypo + R 3 d**	5	79.90±0.51

Hypoglycemia resulted in an increase of brain water content. # p<0.01 compared to Normal Control and Sham Hypo Group. ## p<0.01, compared to Normal Control, Sham Hypo, Sham Hypo + R 24 h (Holm-Sidak method).

Hypo: hypoglycemia, Sham Hypo: sham hypoglycemia. Hypo + R: hypoglycemia + recovery. Data are shown as mean ± SD.

### Detection of brain edema in pathological histology and ultrastructure

**Figure 2:**
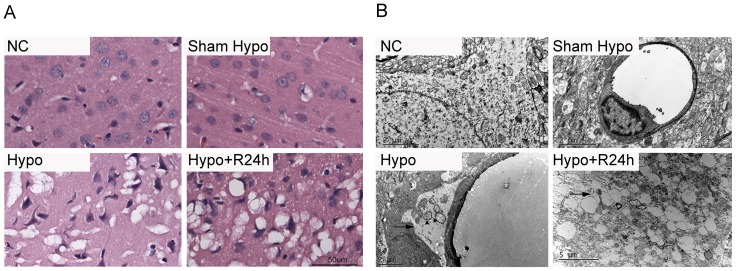
Edematous changes in hypoglycemic rat parietal cortex. A. Hematoxylin and eosin staining showed that vacuolization around neuronal cell bodies and processes was evident after iso- EEG 60 min induced by hypoglycemia (Hypo) and recovery for 24 h(Hypo+R24 h). Nuclear are triangular. B. The end-feet around the capillaries were swollen (arrows), as were the mitochondrion (arrowheads) (Hypo). After 24 h recovery, edema was much more evident around the processes (Hypo+R24 h).

Hypoglycemia-induced brain edema was verified in our histology study. HE staining showed that water accumulated around the cell bodies and diffused to the intercellular space (Fig. 2.A). This was confirmed by TEM, which showed an accumulation of water around the processes. In the end-feet around the vessels, mitochondria were noticeably swollen and cristae had disappeared (Fig. 2.B).

### Late BBB breakdown after hypoglycemia

**Figure 3:**
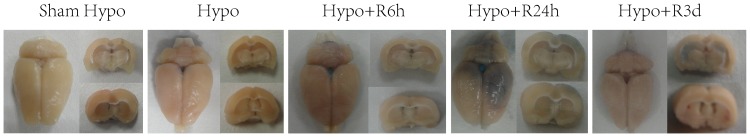
Moderate breakdown of BBB after recovery from hypoglycemia. Evans blue was used to determine the BBB's permeability to plasma protein. No leakage of Evans blue was evident during hypoglycemia but leakage was present 24 h post recovery.

To clarify whether hypoglycemia-induced edema has a vasogenic component, we investigated BBB integrity using Evans blue. Rats from the 24 h recovery group showed increased BBB permeability to Evans blue-albumin complex (Fig. 3). One rat in the hypoglycemia + recovery 3 d group showed hemorrhage in the striatum. All rats in the hypoglycemia group, and the hypoglycemia + recovery 6 h group, had an intact BBB. This suggested that the BBB breakdown occurred during the late recovery period. It should be noted that the asymmetry of the Evans blue leakage may be partly due to the asynchronous onset of iso-EEG between the two hemispheres [2]. This coincides with the sequential phases of brain edema: cytotoxic edema, ionic edema, vasogenic edema, and even hemorrhagic conversion [18]. It suggests that brain edema may be correlated to the period of iso-EEG, and may be more severe after glucose reinfusion. Thus, we cannot exclude the possibility that there may be a vasogenic component to edema.

According to the classic theory, cytotoxic edema develops after extracellular Na^+^ and other cations enter the intracellular space due to a partial failure of energy-dependent mechanisms of extrusion [14]. This can happen during the early stage of a hypoglycemic coma because levels of adenosine triphosphate fall quickly and simultaneously with the onset of iso-EEG [3]. Subsequently, ions and water pass through capillaries under the influence of a gradient induced by cytotoxic edema. This contributes to the increase in brain water content. Previous studies have demonstrated extensive BBB disruption after insulin injection [19]–[21], though these experiments used a higher dose of insulin than our model. Due to the absence of sham hypoglycemia as a control, we cannot exclude the possibility that the large dose of insulin used in previous experiments enhanced BBB permeability. However, in our experiments, the potential effects of insulin on the BBB were eliminated and our conclusion, that there is a late vasogenic element to the development of edema due to endothelial dysfunction, appears reasonable.

### Increased AQP4 and decreased TJ protein expression after hypoglycemia

We measured AQP4 mRNA expression in the cortices of rats subjected to severe hypoglycemia and recovery after hypoglycemia using RT-PCR quantification. A steady increase in AQP4 mRNA expression was detected from the end of iso-EEG 60 min hypoglycemia and during glucose reinfusion compared to that observed in the sham hypoglycemic rats. A significant upregulation of AQP4 mRNA was found in samples from the post-24 h recovery group compared to the sham hypoglycemic animals (p<0.05, Table 2). Even after 3 d, relative expression of AQP4 mRNA was high, though there was a minor decrease compared to the 24 h group (Table 2).

**Table 2:** Results of quantitative RT-PCR analysis of relative AQP4 mRNA expression in hypoglycemic rat brain cortical tissue (mean ± SD).

Group	△C_T_	△△C_T_	2^−△△CT^
**Normal Control**	8.5±1.01	0	1
**Sham Hypo**	8.18±0.32	−0.32±0.32	1.27±0.27
**Hypo**	7.25±0.9	−1.25±0.90	2.75±1.59
**Hypo+R6** **h**	6.63±0.80	−1.87±0.80	3.28±1.15
**Hypo+R24** **h**	6.42±0.56	−2.09±0.56	4.53±1.88^#^
**Hypo+R3 d**	6.42±0.38	−2.08±0.38	4.33±1.11^#^

Levels of AQP4 mRNA were upregulated after 24 h of recovery compared to the Sham Hypo group (p<0.05). # p<0.05 (Dunnett's method, n = 5). CT: cycle threshold for the gene. SD: standard deviation.

**Figure 4:**
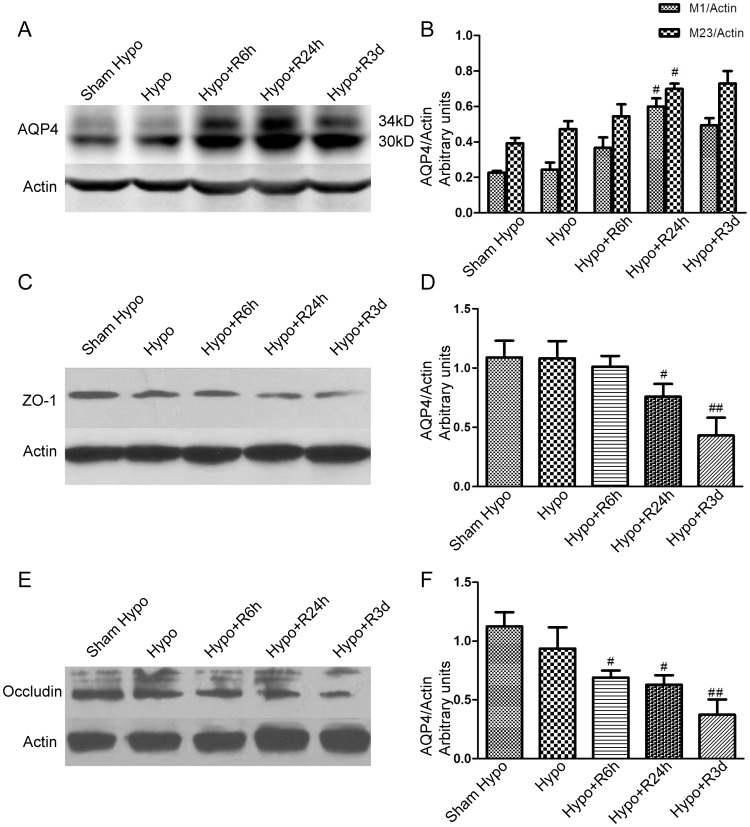
Increased expression of AQP4 protein after hypoglycemia/recovery and decreased expression of tight junction proteins. A. In the representative picture, there are two bands detected for AQP4, corresponding to M1 (34 kD) and M23 (30 kD). B. Quantification of AQP4 expression. Two isoforms both increased with a higher proportion of M1∶M23 in the 24-h recovery group after hypoglycemia versus sham hypoglycemia group. C. Representative picture revealed the decrease of ZO-1 after hypoglycemia. D. Quantification of ZO-1 expression. E. Representative immunoblot of occludin. A significant change of occludin was found after 6 h of hypoglycemia/recovery. F. Quantification of occludin protein expression. # p<0.05,## p<0.01, compared with sham hypoglycemia. (Dunnett's method, n = 5).

Consistent with our described temporal changes in mRNA expression, AQP4 protein expression also increased following hypoglycemia (Fig. 4.A and B). AQP4 is the most abundant water channel in the CNS, and is predominantly located at the borders between fluid compartments and CNS parenchyma, suggesting its importance in transporting water into and out of the parenchyma. Present views suggest that AQP4 may limit the rate of water movement across the BBB during cytotoxic edema, while it may favor fluid elimination during vasogenic edema [22]. Here, we showed that brain edema occurred early in rats in the hypoglycemic group, which was prior to the significant increase in AQP4 expression. These results indicate that AQP4 may not contribute to edema formation.

Our western blot showed two distinct bands (30 kD and 34 kD) that correspond in size to the two isoforms of AQP4. Following hypoglycemia, expression of both isoforms increased significantly in the 24 h recovery group compared to the sham hypoglycemic rats (p<0.05, Fig. 4.B). Increased expression of AQP4 may contribute to increased water permeability. M1 and M23 assemble into heterotetramers that form orthogonal arrays of particles (OAPs). A previous study determined that OAPs are enriched with M23 in the core, and M1 in the periphery [23]. A higher ratio of M23∶M1 results in OAPs of increased size. M1 may block tetramer associations thus reducing the size of OAPs [24]. Increased proportions of M1∶M23 in oocytes results in disorganized OAPs [25]. In this study, we detected that the ratio of M1∶M23 increased with the upregulation of total AQP4 protein expression after hypoglycemia (0.86±0.11 in Hypo+R24 h vs. 0.58±0.09 in Sham Hypo, p<0.05) which may result in disorganization of the OAPs and loss of AQP4 polarization. ZO-1 is a tight junction associated protein. Here, we showed a significant decrease in ZO-1 expression 24 h after hypoglycemia (p<0.05, Fig. 4.C and D). Tight junction proteins, such as occludin, form the BBB. After 6 h of recovery, the expression of occludin protein decreased significantly compared to that in sham hypoglycemia animals (p<0.05, Fig. 4.E and F). However, the biological function of OAPs remains unclear. They may be involved in AQP4 polarization in astrocyte foot processes. Loss of astrocyte polarity has been described in an ischemia model [26] and associated with BBB impairment during experimental autoimmune encephalomyelitis [27]. Disassociation of AQP4 polarization and BBB integrity may explain the late breakdown of the BBB after hypoglycemia. We speculate that the increased proportion of M1∶M23 may affect the formation of OAPs, thus inducing BBB dysfunction. The role of AQP4 polarization in BBB integrity during or following hypoglycemia requires further exploration.

### Immunofluorescence staining showed reactive astrocytes and increased expression and redistribution of AQP4

**Figure 5:**
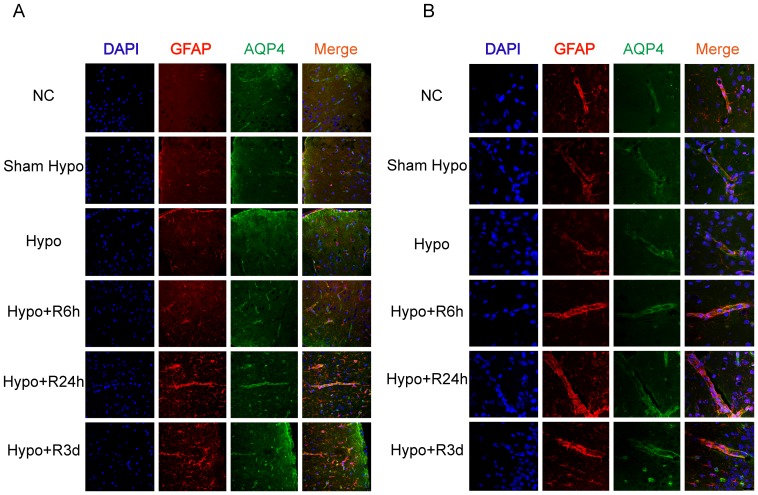
Increased immunereactivity of AQP4 in hypoglycemic rat brain. A. Immunofluorescence staining for AQP4 showed strongest intensity in the rat cortex after 24-hour recovery from profound hypoglycemia. Reactive astrocytes were demonstrated more processes and stronger intensity of GFAP after hypoglycemia shock. B. Immunofluorescence staining for AQP4 showed stronger intensity on the end-feet around cerebral vessels in hypoglycemia group and recovery groups compared with normal control and sham hypoglycemia group.

AQP4 expression in the brain was detected by immunofluorescence staining (Fig. 5.A and B). We showed that AQP4 expression increased significantly in the glial external limitans and end-feet around blood vessels (Fig. 5.B), and was most abundant after 24 h of recovery. Reactive astrocytes were evident after hypoglycemia in the cortex (Fig. 5.A). Edema fluid may be eliminated by three AQP4-dependent routes: through the glia limitans into the subarachnoid cerebrospinal fluid (CSF); through the ependyma into the ventricular CSF; and through the BBB into the blood [28]. Previous research demonstrated that AQP4 could facilitate edematous fluid elimination [29]–[32]. Cytotoxic edema has been tied to necrosis. Intracellular fluid is released from the ruptured membrane and eventually removed by the same route/s as intercellular fluid. In the late recovery period following hypoglycemia, we suggest that the upregulation of AQP4 expression may occur in order to aid in removal of edema fluid.

**Figure 6:**
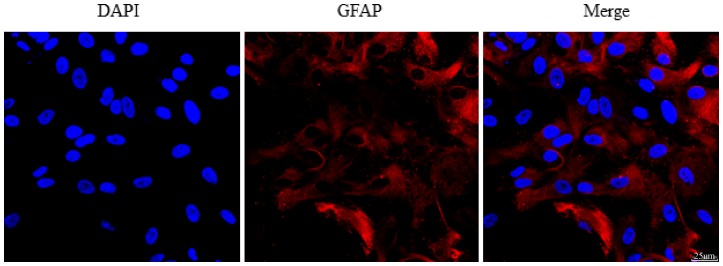
Identification of cultured rat astrocytes by immunofluorescence staining for glial fibrillary acidic protein (GFAP) and 4′,6-diamidino-2-phenylindole (DAPI). About 98% cells are GFAP-positive.

**Figure 7:**
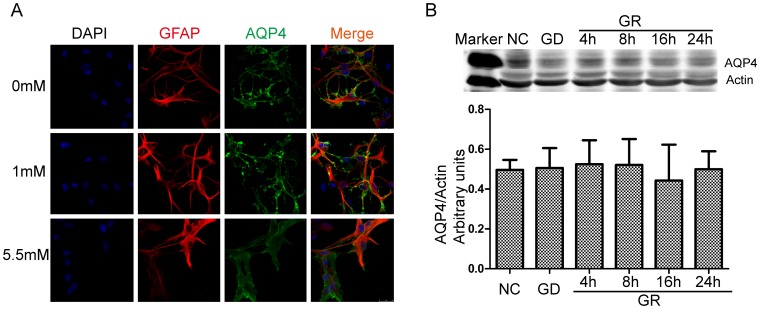
Effect of glucose deprivation on distribution and expression of aquaporin 4 (AQP4) on the plasma membrane of astrocytes. A. After cultured in glucose deficient media for 6 h, astrocytes showed a selective spotted staining of AQP4 on the plasma membrane. B. Western blot analysis of the water channel protein in rat cortical astrocytes that underwent glucose deprivation (GD, DMEM0)/glucose reinfusion (GR, DMEM5.5). No significant change of the total cell protein expression of AQP4 was detected after GD/GR (n = 3, p>0.05). DMEM, Dulbecco's Modified Eagle Medium.

*In vitro* cultured astrocytes were identified by staining with GFAP (Fig. 6). We evaluated the expression and localization of AQP4 in primary astrocyte cultures. Immunofluorescence staining showed that astrocytes cultured in media with a normal concentration of glucose are flat and polygonal, with diffuse AQP4 immunofluorescence in the cells (Fig. 7.A). After glucose deprivation for 6 h, the astrocytes changed and showed a satellite-like morphology with thin processes. AQP4 was detected as obvious, selective spotted staining on the plasma membrane of astrocytes, with little intracellular staining. However, we did not find any change in AQP4 protein expression in our western blot analysis (Fig. 7.B). This is inconsistent with our *in vivo* result. We speculated that this may be due to the absence of hypoglycemia-responsive hormones, such as arginine vasopressin, *in vitro*. Upregulation of AQP4 *in vivo* after hypoglycemia may be not directly regulated by the glucose shortage but mediated by the other systemic pathways that are activated by hypoglycemia.

### GD/GR induced astrocytic injury

**Figure 8:**
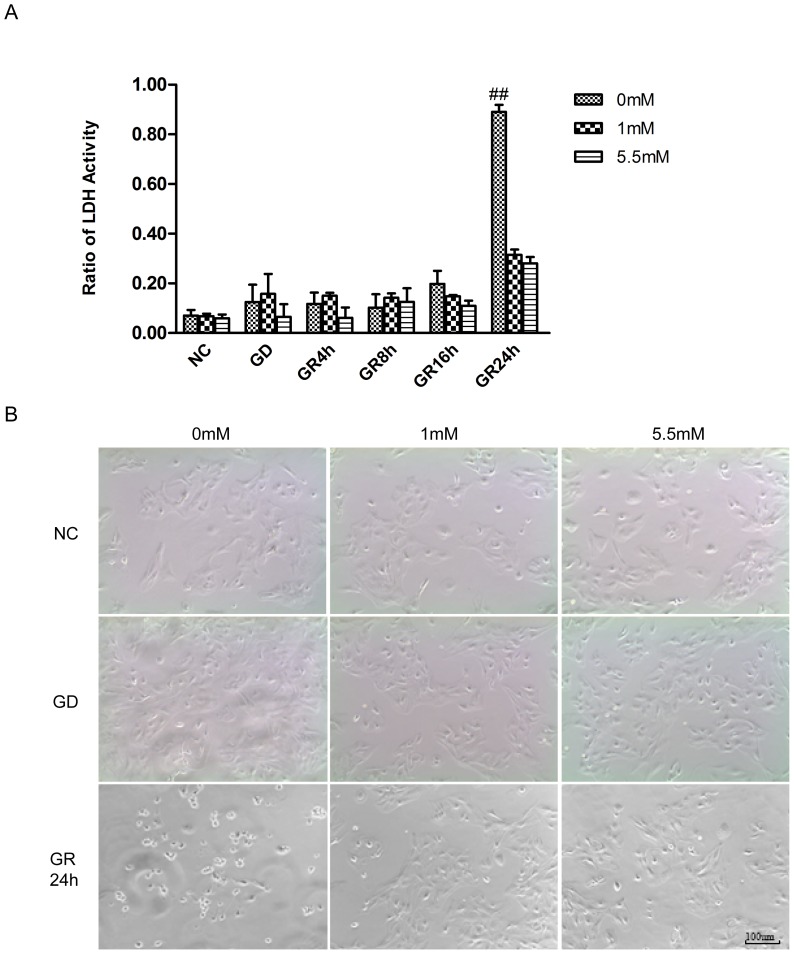
Astrocytic injury induced by glucose deprivation (GD, DMEM0 and DMEM1)/glucose reinfusion (GR, DMEM5.5). A. Quantification of the ratio of LDH activity released in the media to the total cellular LDH showed a significant astrocytic injury in the DMEM0 (0.89±0.03) compared with that in DMEM1 (0.31±0.02) and DMEM5.5 (control, 0.28±0.03, ## p<0.01, n = 3). B. Representative pictures of astrocyte cultures showed clear death of astrocytes after 24 h of glucose reinfusion. DMEM, Dulbecco's Modified Eagle's Medium; LDH, lactate dehydrogenase.

To evaluate the astrocytic injury induced by GD and GR, we measured LDH activity in *in vitro* astrocytes, under conditions that mimicked *in vivo* hypoglycemia. As shown in figure 8.A, the ratio of LDH increased significantly after 24 h of GD/GR (p<0.01). In figure 8.B, the representative images show obvious astrocytic death in DMEM0 24 h after GD/GR. Further study showed that this death was not caspase 3 dependent apoptosis (Fig. S1).

In conclusion, we verified hypoglycemia-induced brain edema in our model and described the expression and redistribution of AQP4 following hypoglycemia. We also found a late breakdown in the BBB after recovery from hypoglycemia. A protective role for AQP4 in maintaining water balance during hypoglycemia needs to be verified. Details of a possible link between AQP4 expression and the BBB breakdown after hypoglycemia remain to be investigated.

## Supporting Information

Figure S1 **GD/GR doesn't induce caspase 3 dependent apoptosis of astrocyte.** Western blotting of caspase 3 and cleaved caspase 3 in astrocytes after GD/GR.
(TIF)
