# Supplementary figures and images for: Expression of Aquaporin 4 and Breakdown of the Blood-Brain Barrier after Hypoglycemia-Induced Brain Edema in Rats
Source: PLoS One. 2014 Sep 29;9(9):e107022. doi: 10.1371/journal.pone.0107022 (PMC4180270; doi:10.1371/journal.pone.0107022)

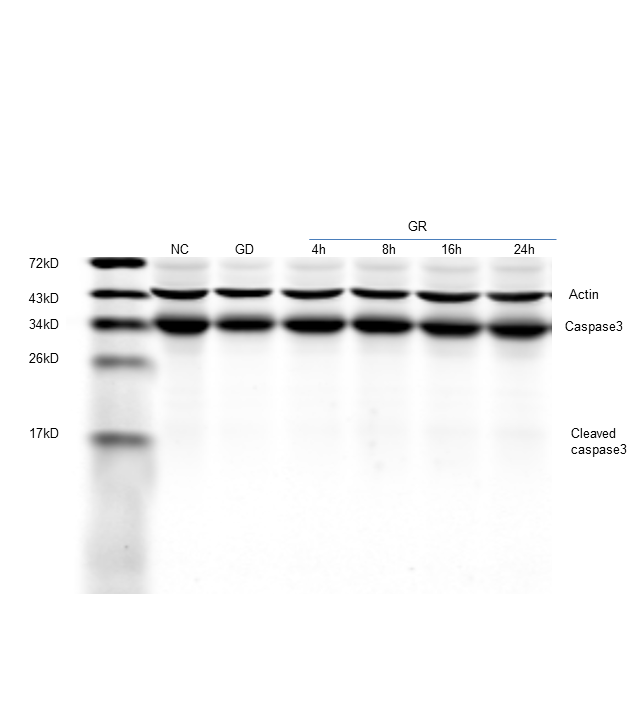

Supplement: Figure S1 — GD/GR doesn't induce caspase 3 dependent apoptosis of astrocyte. Western blotting of caspase 3 and cleaved caspase 3 in astrocytes after GD/GR. (TIF) [file pone.0107022.s001.tif]
